# Production of Bioactive Peptides from Hake By-Catches: Optimization and Scale-Up of Enzymatic Hydrolysis Process

**DOI:** 10.3390/md21110552

**Published:** 2023-10-25

**Authors:** Bruno Iñarra, Carlos Bald, Monica Gutierrez, David San Martin, Jaime Zufía, Jone Ibarruri

**Affiliations:** AZTI, Food Research, Basque Research and Technology Alliance (BRTA), Parque Tecnológico de Bizkaia, Astondo Bidea, Edificio 609, 48160 Derio, Spain; cbald@azti.es (C.B.); mgutierrez@azti.es (M.G.); dsanmartin@azti.es (D.S.M.); jzufia@azti.es (J.Z.); jibarruri@azti.es (J.I.)

**Keywords:** fish, by-product, valorization, proteases, protein hydrolysate, degree of hydrolysis, peptide molecular weight, antioxidant activity, antihypertensive activity

## Abstract

Fish by-catches, along with other fish side-streams, were previously used as raw material for the production of fishmeal and fish oil but appropriate handling allows their use in more valuable options. The aim of this research was to valorize undersized hake (*Merluccius merluccius*) as a model of using fish by-catch from the Bay of Biscay to produce protein hydrolysates with bioactivities. Six enzymes, with different proteolytic activities (endo- or exoproteases) and specificities, were tested to produce protein hydrolysates. Products obtained with an endoprotease of serine resulted in the most promising results in terms of protein extraction yield (68%), with an average molecular weight of 2.5 kDa, and bioactivity yield (antioxidant activity = 88.5 mg TE antioxidant capacity/g fish protein; antihypertensive activity = 47% inhibition at 1 mg/mL). Then, process conditions for the use of this enzyme to produce bioactive products were optimized using Box–Behnken design. The most favorable process conditions (time = 2 h, solids = 50% and enzyme/substrate = 2% with respect to protein) were scaled up (from 0.5 L to 150 L reactor) to confirm laboratory scale and model forecasts. The results obtained in the pilot-scale testing matched the outcomes predicted by the model, confirming the technical viability of the proposed process.

## 1. Introduction

The seafood value chain is of paramount importance in a world with an exponential population growth. In 2019, fish accounted for about 17% of the global population’s intake of animal protein and the total food fish consumption had risen 142% from 1990 to 2019 according to the FAO [1]. However, fish value chain side-streams account for up to 70% of the raw material, due to a combination of policies (discard/by-catch landings), process inefficiencies and the inedible parts of fishes, such as viscera, backbones or heads. The objective of the WaSeaBi project [2] is to unravel the challenges that forestall a more sound exploitation of the aquatic resources. This can be achieved by developing sorting technologies, storage solutions and decision tools to secure an efficient, sustainable supply system for biorefining operations, leading to the valorization of those raw materials into marketable products.

The European Commission Common Fisheries Policy (CFP) introduced a discard ban in 2013 which stated that all catches of species subjected to catch quotas and/or minimum conservation reference size (MCRS) would have to be landed and counted against quota, but at the same time prevented the use of catches under MCRS for direct human consumption [3].

Undersized hake is composed mainly of protein (17%), lipids (between 5–8%) and ash (below 3%), with a moisture content about 75% [4]. The current production from fish by-catches is mainly directed to fishmeal and fish oils; however, this practice may not be the most advantageous or sustainable approach [5]. These fishery by-products could be processed using several hydrolysis techniques such as chemical, enzymatic and/or microbial processing to produce bioactive compounds with different food, feed, biotechnological and nutraceutical applications [6,7,8].

Enzymatic hydrolysis is presented as one of the most interesting processes, breaking peptide bonds in a controlled way and obtaining tailored peptides (poly- and oligopeptides) and free amino acids (fish protein hydrolysates (FPHs)) with different molecular weights required for concrete functional foods and dietary supplements [9]. After applying the hydrolysis process, FPH is separated from the solid part by centrifugation. The remaining solid fraction could be directed to animal feed formulations, while hydrolysates could be tested as functional ingredients in higher-added-value compounds. FPHs rich in bioactive peptides are of special interest for the food sector as they could promote health [10]. Bioactive peptides are composed of 3 to 20 amino acids and their mode of action rests on the amino acid composition and the amino acid sequence [11]. The main bioactivities that have been described in the literature are antioxidant, antimicrobial, antihypertensive, anticancer, anticoagulant and calcium-binding activity [12].

In recent times, there has been a growing necessity to enhance the shelf life of food products through the use of antimicrobial agents, with synthetic options being the most prevalent choice, mainly due to a higher level of global concern over foodborne illnesses [13]. However, there is a rising demand among consumers for more natural components in their daily life. This trend has increased interest among researchers to explore natural alternatives [14]. Similarly, there is a significant consumer interest in antioxidant compounds, as the consumption of foods rich in antioxidants has been linked to various health benefits, including the reduction of cardiovascular diseases [15], while also contributing to the preservation of food products. Additionally, hypertension is also becoming a concern among people, causing 10.4 million deaths in 2019 [16], and it is predicted to affect up to 1.5 billion by 2025 [17].

The aim of this research was to produce FPH with bioactivities by using undersized hake (*Merluccius merluccius*) as a model of fish discard from the Bay of Biscay. These by-catches, along with other fish side-streams, were previously used as raw material for the production of fishmeal and fish oil but appropriate handling may allow their use in more valuable products. The objective of the present study was to pre-screen the potential of six enzymes to produce FPH and to optimize the process conditions (time, enzyme % and solids %) of the most promising enzyme in terms of yield and bioactivity. In addition, the optimized process was scaled up in order to test the technical feasibility of producing bioactive peptides on a larger scale.

## 2. Results and Discussion

### 2.1. Initial Screening: Enzyme Selection

Table 1 shows the characteristics of the used enzymes and the process conditions employed in each screening run.

**Figure 1:**
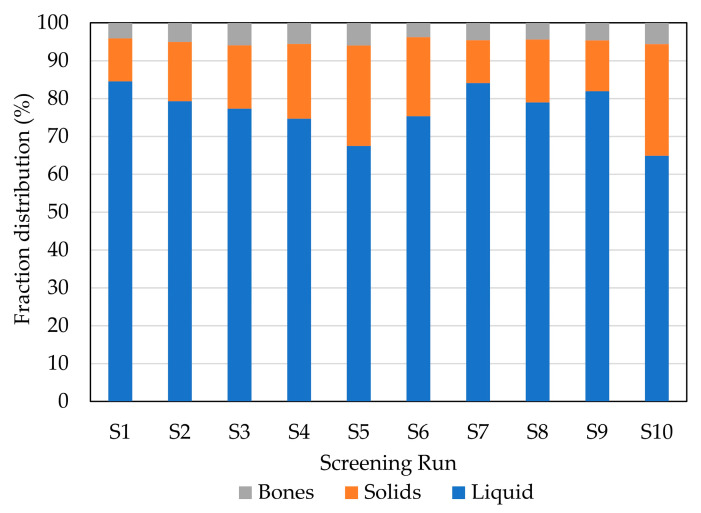
Fractions distribution in different enzymatic hydrolysis runs of the initial screening. For screening experimental design conditions, see Table 1.

**Figure 2:**
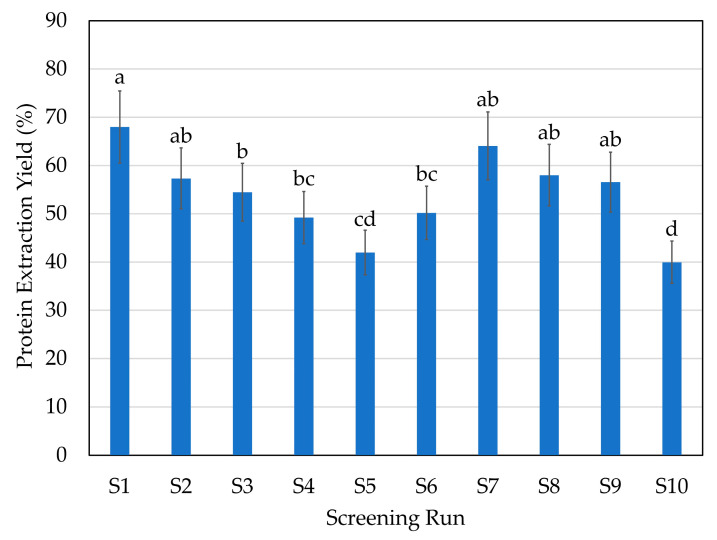
PEY (protein extraction yield, %) in the different enzymatic hydrolysis runs of the initial screening. Error bars represent SD (*n* = 3). Same letter means no significant difference between samples at 95% confidence. For screening experimental design conditions, see Table 1.

Hydrolysis processes with the different enzymes yielded 67.5–84.6% fish protein hydrolysate (FPH, water-based fraction), 11.3–26.5% solid fraction and 3.8–6.0% bone fraction (Figure 1). The highest hydrolysate yield (highest recovery of FPH) was obtained with enzyme A (endoprotease of the serine type) at 70 °C (run S1) and with those enzymatic processes where a combination with exopeptidases (F or G enzymes) was used (S7, S8, S9). As expected, the control run (CTR, run S10) led to the lowest liquid recovery (64.9%) and highest solid recovery (29.5%). The FPH contained from 6.1 to 7.5% protein, which implies a 40–65% protein extraction yield (PEY) (Figure 2). The highest PEY was achieved with enzyme A at 70 °C (S1), followed by S7 (combination of enzymes A and F), S8 (combination of enzymes P and F), S2 (enzyme A at 50 °C), and S9 (combination of enzymes P and G), although there were not statistically significant differences. Tadesse et al. [18] also found a higher PEY using endoprotease of serine type, due to its higher hydrolytic activity per mL of sample. The addition of the F enzyme, being a mixture of endo- and exopeptidases, usually increases the hydrolytic capacity of the process, leading to higher extraction yields [19]. Predictably, the CTR (S10) led to the lowest PEY, which is related to the non-addition of enzymes. However, the endogenous fish enzymes activity resulted in a PEY of almost 40%.

**Figure 3:**
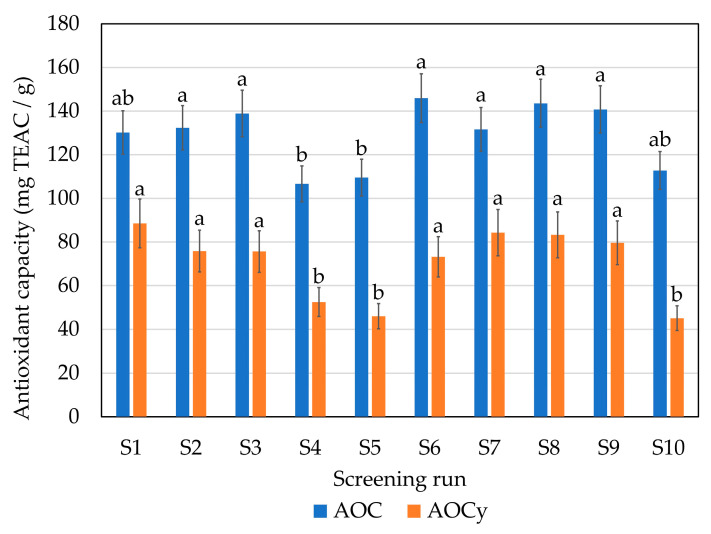
Antioxidant capacity (AOC) and antioxidant capacity yield (AOCy) in the different enzymatic hydrolysis runs of the initial screening. Error bars represent SD (*n* = 3). Same letter means no significant difference between samples in the same series at 95% confidence. For screening experimental design conditions, see Table 1.

Regarding antioxidant activity (AOC), all the produced hydrolysates showed high AOC with 107–144 milligrams of Trolox equivalent antioxidant capacity (TEAC) per gram of protein, with the most active being those produced with S1 and S2 (endoprotease of the serine type), S3 (broad-spectrum endoprotease), S6 (glutamic acid-specific protease), S7 (endoprotease of the serine type + blend of endo- and exopeptidases), S8 (broad-spectrum endoprotease + blend of endo- and exopeptidases) and S9 (broad-spectrum endoprotease + glutamic acid-specific protease) (Figure 3). In the case of CTR (S10), the entire fish was processed, including the digestive system where the fish’s own endogenous enzymes are found; in consequence, a relatively high AOC was observed (113 mg TEAC/g protein). Antioxidant activity in protein hydrolysates may depend on several factors: the type and structure of the peptides, their amino acid composition and the enzyme and the by-product composition [20]. Some studies have suggested that ABTS (2,2-azinobis-(3-ethylbenzthiazoline-6-sulfonic acid)) radical scavenging activity is related to the molecular size of the peptide and that may be independent of the protease type [21]. Furthermore, an increase in the degree of hydrolysis (DH) has been reported to increase ABTS radical scavenging activity in different matrices, such as gelatine [22] or tilapia [23].

**Figure 4:**
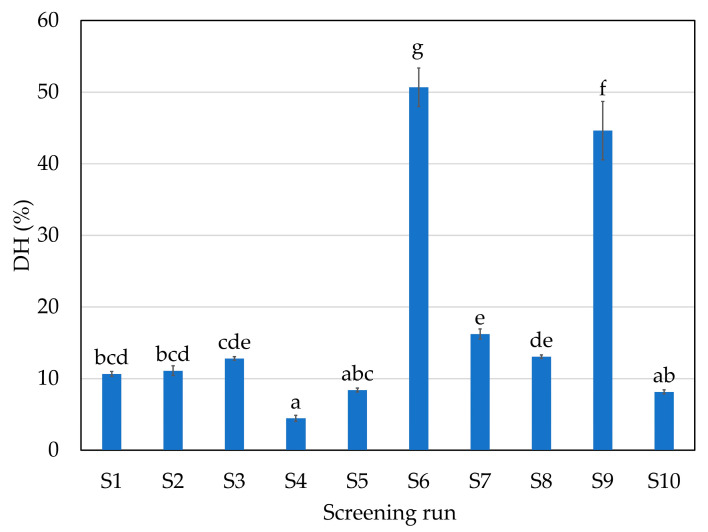
Degree of hydrolysis (%) of the liquid fraction in the different enzymatic hydrolysis runs of the initial screening. Error bars represent SD (*n* = 3). Same letter means no significant difference between samples in the same series at 95% confidence. For screening experimental design conditions, see Table 1.

Regarding antioxidant capacity yield (AOCy), expressed as total mg of TEAC/g of processed protein (initial protein calculated in the hake), however, this was higher with enzyme A at 70 °C (S1). No differences were found between the S1, S2, S3, S6, S7, S8 and S9 runs. The lowest values were obtained with S4 (trypsin-specific protease) and S5 (chymotrypsin-like protease) and with the S10 (CTR); those screening runs also resulted in the lowest DH (Figure 4).

In the present study, higher DH hydrolysates showed higher antioxidant activity and the lowest DH hydrolysates showed lower antioxidant activity. The DH is the number of peptide bonds that are cleaved during the hydrolysis process and it gives an idea of the average size of the peptides present in the FPH. Furthermore, it is also related to the percentage of protein recovery yield and resulting bioactivities [24]. The inclusion of the exopeptidase F increased the DH from the screening runs S1 and S2 to S7 (Figure 4); however, a statistically significant increase was not observed in the antioxidant capacity (Figure 3). Furthermore, the S6 and S9 runs produced hydrolysates using glutamic acid-specific protease and led to a statistically higher DH (between 45–55%) compared to the other runs. Hake protein is composed of a high percentage of glutamic acid (11.7% of total amino acids) [4]; thus, the use of a specific protease could lead to higher DH.

Although the DH increased, antioxidant capacity was not increased proportionally in those cases, mainly because, in addition to the DH, the composition and distribution of the amino acids within the peptide chain largely determine the antioxidant capacity of the peptides. Consequently, the main variations in antioxidant activities with similar DH may be mainly due to both the composition and the distribution of amino acids in the peptide chain [25]. Chi et al. [26] also reported that low MW fractions have more donating electron/hydrogen peptides that could interact with free radicals and therefore stabilize the products. Nalinanon et al. [27] obtained ornate threadfin bream (*Nemipterus hexodon*) muscle hydrolysate using skipjack tuna pepsin, with antioxidant activity (159 μmol Trolox equivalent/g protein) determined by the ABTS method, that corresponded to 39.75 mg Trolox equivalent/g protein, a lower value than the ones obtained in our FPHs (Figure 4). Other authors also found lower values than the ones reported in this study, from 2 to 25 mg Trolox equivalent/g protein in FPHs obtained from samples of grenadier (*Macrourus* sp.), megrim (*Lepidorhombus boscii*), European hake (*Merluccius merluccius*), boarfish (*Capros aper*) and Atlantic horse mackerel (*Trachurus trachurus*) skin, head and bones. Several authors tried to optimize and maximize the production of antioxidant compounds from FPHs. As examples, Taheri et al. [28] isolated different peptide fractions from herring brine by ultrafiltration to evaluate their bioactivity potential, while Vázquez et al. [29] analyzed the pH and temperature in the hydrolysis of *S. canicula* muscle by three commercial proteases using response surface methodology (RSM).

**Figure 5:**
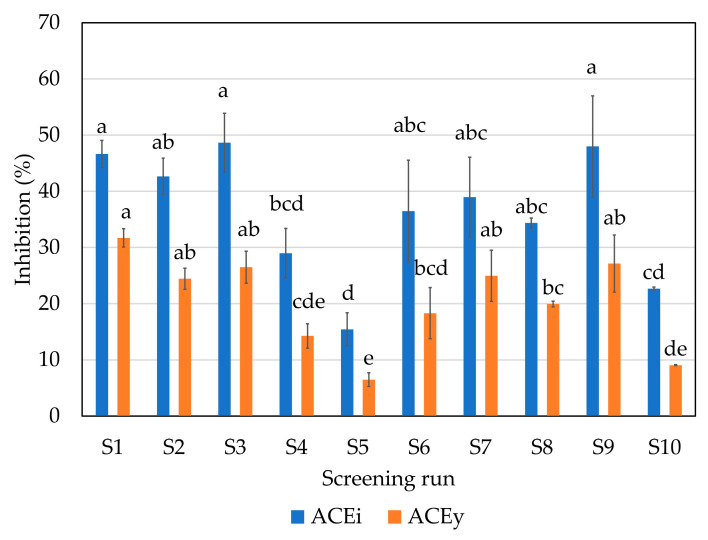
Antihypertensive activity expressed as ACEi (angiotensin-converting enzyme inhibition) at 1 mg/mL and ACEy (antihypertensive capacity yield) in the different enzymatic hydrolysis runs of the initial screening. Error bars represent SD (*n* = 3). Same letter means no significant difference between samples in the same series at 95% confidence. For screening experimental design conditions, see Table 1.

The antihypertensive activity of an FPH is also related to its DH. As happens with the AOC, a higher DH led to higher angiotensin-converting enzyme inhibition (ACEi, Figure 5). The highest values (between 42.6 and 48.0% inhibition) were found in runs S1, S2, S3 and S9 (endoprotease of serine type at 70 and 50 °C, broad-spectrum endoprotease and broad-spectrum endoprotease + glutamic acid-specific protease, respectively). The lowest values otherwise were found in CTR (S10), S4 and S5 (trypsin-specific protease and chymotrypsin-like protease). The ACEi activity depends on the substrate hydrolyzed and the corresponding MW profiles of FPHs. Similar inhibition values were obtained between 42 and 45% of ACEi at a concentration of 1 mg protein/mL using skin and bones and heads of hake [5].

Vázquez et al. [30] also found ACEi values that varied from 53% to 82% with the maximum response in turbot viscera hydrolysates using Alcalase 2.4 L enzyme. Other authors also found values in the same inhibition range [31,32,33,34].

Antihypertensive capacity yield (ACEy) shows the inhibition effect in terms of protein yield. As expected, the highest value was obtained using enzyme A at 70 °C (the conditions that led to the highest PEY), followed by the S2, S3, S7 and S9 runs. The lowest values otherwise were shown in the S5 run (chymotrypsin-like protease enzyme) followed by the S10 (CTR).

Considering that the hydrolysis with enzyme A (endoprotease of the serine type) at 70 °C led to the best results in terms of PEY and bioactivities, it was selected for further process optimization.

### 2.2. Hydrolysis Optimization

The experimental responses for each variable—PEY (%), DH (%), AOC (mg TEAC/g protein), AOCy (mg TEAC/g fish protein), ACEi (% inhibition at 1 mg protein/mL), ACEy (% inhibition at 1 mg fish/mL)—of each hydrolysis condition included in the experimental design are shown in Table 2.

The experimental responses were fitted to a polynomial model to assess the effect of the variables on the response. The insignificant coefficients for the full quadratic model and their significance levels were analyzed. Since there were insignificant terms, the model was reduced by removing them using a *p*-value of 0.9 as the cut-off. Table 3 shows the coefficients and the statistical significance (*p* value) of the reduced model.

The fitted model goodness was assessed by the coefficient of determination (R^2^) and the R^2^ adjusted, which are shown in Table 3. The highest values for R^2^ and adjusted R^2^ were for the PEY (R^2^ 94.10 and Adj. R^2^ 88.16), DH (R^2^ 85.70 and Adj. R^2^ 59.96), AOCy (R^2^ 86.76 and Adj. R^2^ 62.93) and AOC (R^2^ 79.65 and Adj. R^2^ 52.52). The high values of R^2^ for the models indicated that there was a good correlation between the experimental and predicted response values. On the contrary, in the case of the prediction of ACEi and ACEy, the obtained R^2^ and the R^2^ adjusted were lower (R^2^ 61.44 and Adj. R^2^ 10.02 for ACEi and R^2^ 72.76 and Adj. R^2^ 45.51 for ACEy), revealing a worse relation between the experimental and predicted values.

Regarding the results obtained during the optimization process, PEY varied from 29.36 to 61.64%, and the ANOVA analysis showed that four variables had a statistically significant effect on the results within the studied limits: the % of solids (with a negative effect), time (with a positive effect), the enzyme concentration (with a positive effect) and the quadratic term of % of solids.

The DH of the extracted proteins values varied between 4.32 and 12.64% and only the process time of hydrolysis showed a sensitive response within the studied range, with a positive effect. This fact may be related to the enzymes present in the fish itself, which, since they were not previously inactivated, had a major effect on the DH, as shown in Figure 3. Thus, the factor with the greatest impact was the time factor.

Concerning bioactivities, AOC resulted in values between 125 and 191 mg TEAC/g protein, and two factors had a significant effect: the enzyme concentration increased the AOC, while the interaction between enzyme concentration and time showed a negative effect. ACEi values were between 6.74 and 33.59% of inhibition at a concentration of 1 mg/mL and none of the factors had significant effects. In the case of bioactivity yields, AOCy was only affected by enzyme concentration, with a positive effect, and ACEy was affected by hydrolysis time (negative effect) and a positive effect from enzyme concentration (Table 3).

Optimization techniques are usually required to determine the most favorable working conditions to produce the added-value ingredients. In this regard, several authors have used RSM and experiment design as key elements for selecting optimum process conditions during FPH production [18,25,35]. As happened with our results, Korkmaz et al. [35] found that the DH was not affected by enzyme concentration (alkaline protease) in the hydrolysis of trout and anchovy waste, but the DH was affected in Blue Whiting waste hydrolysis. However, when using flavourzyme, the effect of enzyme concentration was significant in increasing the DH of trout waste hydrolysates, leading to the conclusion that the most important process parameters to increase the DH in FPH are the fish species and composition, enzyme type and hydrolysis method.

In a general sense, PEY increases as enzyme percentage increases, principally because there are more enzyme molecules to associate to the substrate, cleaving larger numbers of peptide bonds and releasing more soluble proteins [36]. In our case, enzyme concentration (the higher the dose, the higher the PEY) and time (the longer the time, the higher the DH and the higher the PEY) had a significant effect on the PEY. The solids concentration, however, had a significant negative effect. The same effect was obtained in an FPH obtained from *Labeobarbus nedgia* using Alcalase^®^ and Novozym^®^ enzymes [18].

Morales-Medina et al. [25] analyzed the effects of the percentage of two enzymes (subtilisin and trypsin), substrate concentration and temperature on the antioxidant activity of FPHs, measured by three different methods (DPPH scavenging activity, Fe^3+^ reducing power and iron (Fe^2+^) chelating activity). They concluded that the best process parameters were not the same for all the bioactivities and that some of the experimental conditions that led to an optimal activity in one of the antioxidant action methods could affect negatively the others.

In this study, a similar conclusion was reached, because when different optimization objectives were set, the optimal process conditions varied (Table 4). If the process focused on PEY, the resulting model indicated that process conditions must be set at Solids: 50.0%; Time: 3.07 h; Enzyme 2.0%. However, if we focused on the bioactivities maximization of both AOC and ACEi, we obtained different values: Solids: 65.0%; Time: 2.0 h; Enzyme 2.0% for AOC, and Solids: 50.0%; Time: 2.0 h; Enzyme 2.0% for ACEi. Therefore, one must decide between maximum peptide yield and maximizing their bioactivity. To address this issue, the AOCy and ACEy were evaluated and optimized, leading to the same process conditions (Solids: 50.0%; Time: 2.0 h; Enzyme 2.0%), so that one could obtain the best hydrolysis results in terms of higher bioactivity and bioactive compound yields.

**Figure 6:**
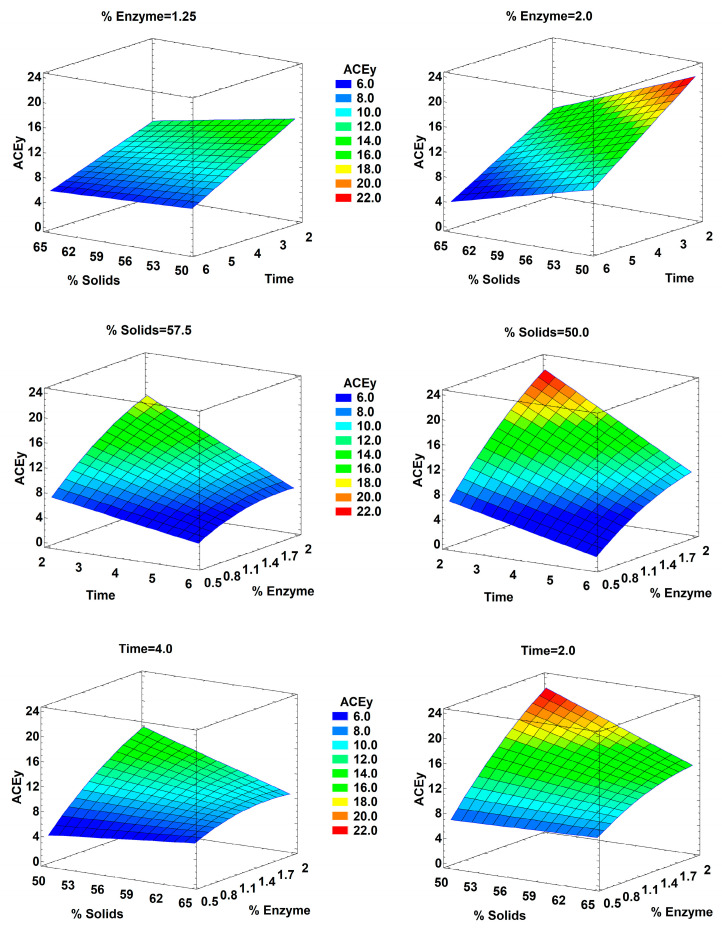
Response surface plots of the antihypertensive capacity yield (ACEy % at 1 mg/mL) for mean parameter values (**left column**) and for maximum antihypertensive capacity conditions (**right column**).

Response surface plots were analyzed in the responses with significant factors. Figure 6 shows the response surface plots of the ACEy variable. Surfaces showed that a decrease in time (near to 2 h) was favorable for the production of ACEi activity peptides. In the case of solids concentration, the effect of this parameter on the ACEy was dependent on other variables due to interactions. For example, at 1.25% of enzyme, the solids concentration had less effect on the ACEy variable, whereas at 2% of enzyme, decreasing the solids from 65% to 50% almost doubled the ACEy value. In general, the combinations of higher enzyme concentration and lower incubation period, and lower solids concentration and higher enzyme concentration, led to higher ACEy.

**Figure 7:**
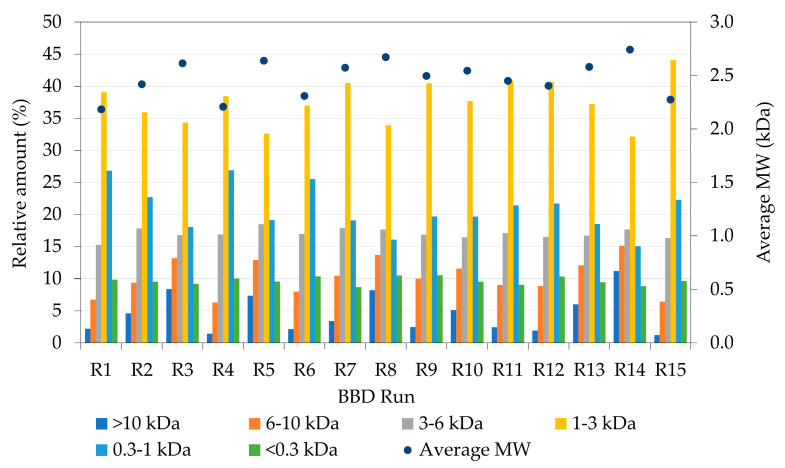
Relative amount (%) of molecular weight fractions and average molecular weight (MW) in each run of the Box–Behnken design (BBD).

Figure 7 shows the MW profile of the hydrolysates of the BBD. The mean MW went from 2.18 to 2.74 kDa, with the most abundant fraction being the one between 1 and 3 kDa (with a mean relative abundance of 37.7%). Bougatef et al. [34] also found similar MW fractions in their main peptides with values between 1.23 and 1.58 kDa. Other authors [30,37,38] also found similar mean MW values for their FPHs. Rodrigues et al. [37] found an FPH with an average MW of 1.71 kDa from Atlantic codfish frames using Alcalase enzyme with a relative abundance of 41% in the range of 1–3 kDa, and Vázquez et al. [30] found mean MW values from 1.2 to 2.14 kDa.

Antioxidant and antihypertensive bioactivities depend on several factors (initial substrate, enzyme, amino acid sequence, peptide size, etc.), as explained above. One of the main factors is the MW profile of the FPH. In this regard and in order to analyze the existing correlation between the different ranges of molecular weights and bioactivities, a univariate correlation between the variables was performed.

**Figure 8:**
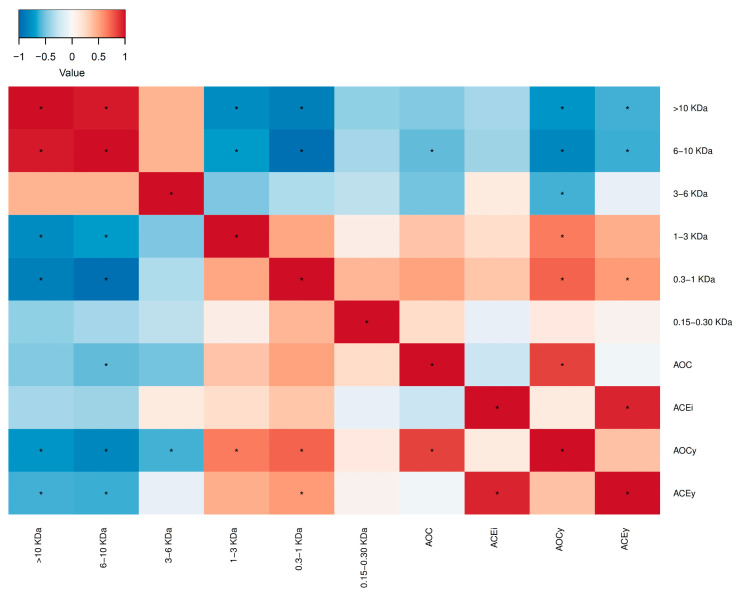
Spearman correlation heatmap; red indicates a correlation of 1, blue −1. The color gradient illustrates the strength and direction of the correlation. Asterisks (*) denote statistically significant values (*p* < 0.05). AOC: antioxidant capacity; AOCy: antioxidant capacity yield; ACEi: antihypertensive capacity; ACEy: antihypertensive capacity yield.

Figure 8 shows the correlations between the MW fractions and bioactivities (AOC and ACEi) and bioactivity yields (AOCy and ACEy). A statistically significant positive correlation was found between low MW fractions (0.3–1 kDa) and bioactivity yields. Moreover, AOCy and ACEy were negatively correlated with higher MW fraction (higher than 3 kDa in the case of AOCy and higher than 6 kDa in the case of ACEy). However, the bioactivities (AOC and ACEi) presented lower correlations with statistical significance and only the AOC presented a negative correlation with the molecular weight fraction between 6 and 10 kDa. A higher correlation between the different peptide fractions and the yields of bioactivities was due to the fact that these parameters are multiplied by the PEY, which in turn is positively correlated to the lower MW fractions, since the higher the peptide release, the higher the protein extraction yield. Vázquez et al. [30] concluded that peptides with a MW slightly higher than 1 kDa (between 1.3 and 1.8 kDa) obtained from turbot filleting presented higher antioxidant and antihypertensive bioactivities. Such findings were also obtained by other authors using a wide variety of fish substrates (cod, tilapia, tuna) and analyzing a wide range of bioactivities (ACE, cellular oxidative stress, antioxidant, etc.) [39,40,41]. Other authors [42], on the other hand, concluded that peptides with low MW (<1 kDa) and shorter chain length (<20 amino acids) exhibited higher antihypertensive activity. Some authors improved their FPHs by applying separation and concentration techniques such as size exclusion chromatography. As an example, García-Moreno et al. [43] increased bioactivities through the isolation of specific MW peptide fractions (a fraction between 0.5–1.2 kDa and a fraction lower than 0.5 kDa). Therefore, these types of techniques are of great help to know which are the fractions with the highest bioactivity and to proceed to their concentration and purification.

### 2.3. Hydrolysis Scaling-Up

**Figure 9:**
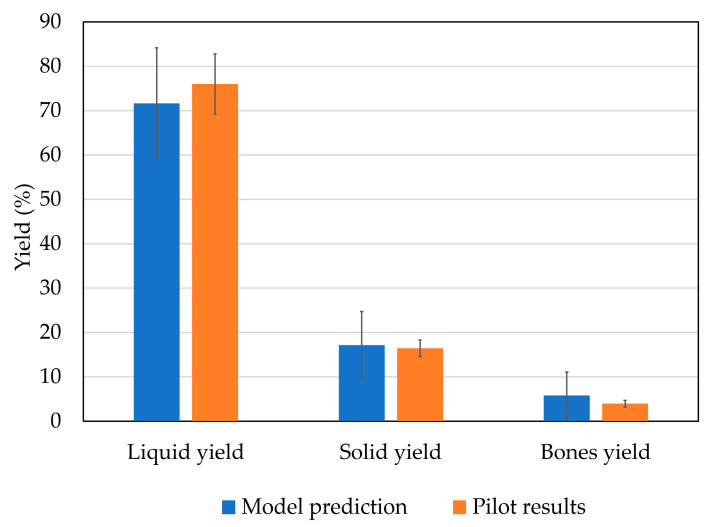
Comparison between model-predicted and pilot hydrolysis yield results.

The optimum conditions were used to produce bioactive peptides at pilot level and to compare the values predicted by the model with those obtained at pilot scale. Resulting fraction yields in the pilot test did not present any statistical differences from the results predicted by the model (Figure 9). Liquid yields were 76.0 ± 6.8%, solid yields 16.4 ± 1.9% and bones yields 3.9 ± 0.7%, with respect to the initially processed weight.

**Figure 10:**
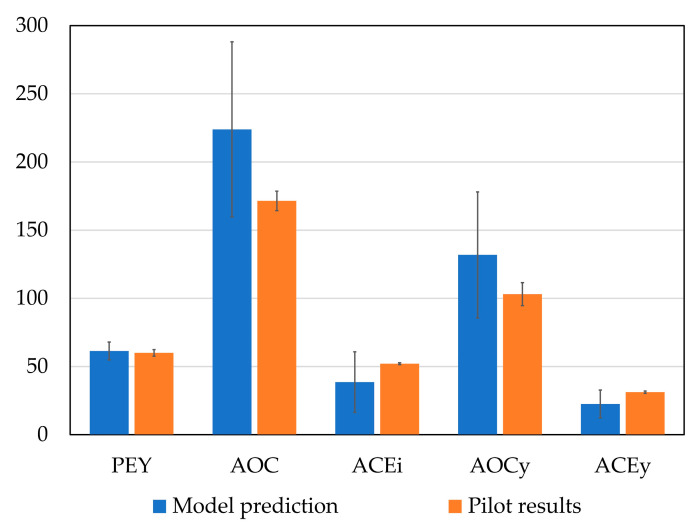
Comparison between model-predicted and pilot hydrolysis results in protein extraction yield (PEY, %), antioxidant capacity (AOC, mg TEAC/g), antihypertensive capacity (ACEi, % inhibition at 1 mg/mL), antioxidant capacity yield (AOCy, c mg TEAC/g) and antihypertensive capacity yield (ACEy, % at 1 mg/mL).

Resulting pilot products had similar PEY (60.0 ± 2.4% pilots and 61.4% predicted), slightly lower AOC (171.5 ± 7.1 mg TEAC/g protein in pilots and 223.9 mg TEAC/g protein predicted) and AOCy (103.1 ± 8.4 mg TEAC/g protein in pilots and 131.9 mg TEAC/g protein predicted), and higher ACEi (52.1 ± 0.7% inhibition in pilots and 38.6% predicted) and ACEy (31.2 ± 0.9% inhibition in pilots and 22.6% predicted); however, all the results were within the predicted model deviation and therefore there were no statistically significant differences (Figure 10).

The production of FPHs from hake by-catches at the pilot plant scale obtained similar results compared to lab experiments, confirming the technical viability of the proposed process.

Even though fish by-products contain numerous nutrients and bioactive compounds with the potential to enhance their market value, they are primarily employed in the production of fishmeal for animal feed. Achieving sustainable fisheries necessitates the adoption of innovative technologies and processes to facilitate the utilization of these by-products for the production of value-added compounds. In this context, enzymatic hydrolysis represents a method for controlled release of these compounds, opening up opportunities for applications in the food, cosmetic, nutraceutical and pharmaceutical industries. Such developments would contribute to the sustainability of all involved sectors.

## 3. Materials and Methods

### 3.1. Raw Material

Undersized hakes, as a model of fish by-catch (also known as discard, ex-discard or unwanted catches in the bibliography) from the Basque fleet, were collected in Ondarroa (Basque Country, Spain) from a bottom trawler in October 2022. Once landed, the samples were directly brought to AZTI’s facilities, packed in vacuum in small quantities and frozen at −20 °C until used.

Prior to processing, the samples were thawed at 5 °C and the whole fish was slightly grinded to homogenize the side-stream.

### 3.2. Enzymatic Hydrolysis at Laboratory Scale

The hydrolysis processes for the screening and the optimization trials were performed at laboratory scale using Symphony 7100 Bathless Dissolution Distek equipment (Distek Inc., North Brunswick, NJ, USA). In all the processes, the temperature, pH, time and stir speed were controlled and monitored. After the hydrolysis process, enzymes were inactivated by heat treatment at 95 °C for 15 min. Then, the content of the vessel was sieved to separate the bones, and centrifugated (2650× *g*; 15 min; ambient temperature) to separate 2 different layers: (1) a water-based fraction (the fish protein hydrolysate, FPH) and (2) a solid pellet. The amount of fat recovered in the hydrolysis processes was neglectable. FPHs were freeze dried for further processing. All the hydrolysis processes were carried out in triplicate.

#### 3.2.1. Initial Screening

Six enzymes with different enzymatic activity were tested to produce FPH: a broad-spectrum endoprotease (P), an endoprotease of the serine type (A), a trypsin-specific protease (T), a chymotrypsin-like protease (C), a blend of endo- and exopeptidases (F) and a glutamic acid-specific protease (G). A control (CTR) was added to the trials and was performed without enzyme addition at pH 6.0, 50 °C and 3 h.

The pH of each run of the experimental design was controlled manually and adjusted with NaOH 1 M (Fischer Scientific, Loughborough, UK) in a final mass of 500 g (250 g of side-stream + 250 g water, referred to as 50% solids). All the processes were carried out with 1% enzyme/substrate (concentration calculated based on the protein content, or substrate, in the reaction vessel), for 3 h at 250 rpm and at the optimum pH and temperature for each enzyme or enzyme combination (Table 1).

#### 3.2.2. Optimization via Box–Behnken Design (BBD)

With the aim of optimizing the hydrolysis parameters for the selected enzymes, a Box–Behnken design (BBD) was carried out. The BBD was composed of three factors and three levels to fit a second-order model. The selected factors for the hydrolysis process were enzyme (in weight basis)/substrate (protein) ratio (A, 0.5–1.25–2%), solids (B, 50–57.5–65%, calculated as grams of raw material in the total mass of the vessel content) and time (C, 2–4–6 h), and their effects on protein extraction yield (PEY, %), degree of hydrolysis (DH, %), antioxidant capacity (AOC), antioxidant capacity yield (AOCy), antihypertensive capacity, measured as angiotensin-converting enzyme inhibition (ACEi), and antihypertensive capacity yield (ACEy) were analyzed.

A final quantity of 500 g in the vessel (250 g water + 250 side-stream), a 70 °C process temperature and pH 9 were used in all the runs.

The experimental design comprised 15 trials, including three replicated center points (see Table 2).

The central data points facilitate the assessment of pure error and system performance at any experimental point within the examined range [44]. Hydrolysis procedures were systematically conducted in a randomized order to mitigate potential bias.

Results were expressed as a second-order polynomial equation, as shown in Equation (1):

Yij = A0 + ∑AiXi + ∑AiiXi^2^ + ∑AijXiXj + ε (1)

where Yij is the response function, A0 is the regression coefficient for the intercept, Ai is the coefficient of the linear term, Aii is the coefficient for the quadratic term, Aij is the coefficient of the interaction term, and ε is the error.

### 3.3. Process Scale-Up

The optimized process was scaled up in a 150-liter stirred reactor (Bachmix-L 150, Bachiller, Parets del Vallés, Spain) to assess the product’s reproducibility at larger scale at the optimized hydrolysis conditions (50% of solids, enzyme substrate ratio 2% and time 2 h). Process temperature and pH were maintained at 70 °C and 9, respectively. Previous to the hydrolysis process, the undersized hakes were minced using a multipurpose grinder (Bio-multipurpose grinder BG2–505 KW Voran, Pichl bei Wels, Austria) and were added to the reactor once the water was at the desired temperature (70 °C). Then, the mixture was heated up to process temperature (70 °C), the pH was adjusted to 9 using NaOH (10 M) and the enzyme was added. After the hydrolysis time (2 h), the enzyme was inactivated by increasing the temperature in the reactor up to 95 °C for 15 min. The resulting hydrolysate was sieved in a 0.5 mm sieve to recover the bones and centrifuged in a Clara 20 Concentrator (Alfa-Laval, Lund, Sweden) to obtain a liquid (FPH) and a solid fraction (100 l/h, 11,000× *g*, solid discharge each 3 min). The amount of fat recovered in the hydrolysis processes was neglectable. The FPHs were freeze dried and the other co-products (bones and solids) were frozen (−20 °C) until further processing.

### 3.4. Analytical Determination

If not specified, the manufacturer of the chemical reagent used was Sigma-Aldrich (Steinheim, Germany). All the analyses were carried out in triplicate.

#### 3.4.1. Chemical Analysis

The chemical composition of the samples was measured by applying the Association of Official Analytical Chemists (AOAC) *Official Methods* [45], dry matter (method 934.01) and nitrogen (method 984.13), using a nitrogen-to-protein conversion factor of *n* × 6.25.

Protein extraction yield (PEY %) was determined as follows in Equation (2):

PEY (%) = (Protein content in the hydrolysate, g)/(Protein content in the initial sample, g) × 100(2)

where “protein content in the hydrolysate” was calculated by multiplying the weight of the hydrolysate by the protein concentration measured by the Kjeldahl method and the “protein content in the initial sample” was calculated by multiplying the weight of the initial sample by the protein concentration measured by the Kjeldahl method.

#### 3.4.2. Degree of Hydrolysis

The O-phthaldialdehyde (OPA) method was applied to quantify the degree of hydrolysis (DH %) [46]. Briefly, a 3.7% *w*/*v* Na tetraborate decahydrate and 0.58% *w*/*v* Nadodecyl-sulphate aqueous solution was mixed with 4% (*w*/*v*) methanolic solution of OPA in a proportion of 51.5:1. Then, 0.38% *v*/*v* of mercaptoethanol was incorporated into the solution. The sample solutions were standardized to 0.01 g protein/L. A total of 60 µL of sample and 180 µL of OPA reagent were dosed in microplate wells in a method adapted from Tejano et al. [47]. They were incubated at ambient temperature for five minutes. The determinations were carried out at 360 nm excitation wavelength and 460 nm emission wavelength. *n*-Acetyl-L-lysine was used as standard. The DH was calculated as described by Nielsen et al. [46]. Htot, defined as the total number of peptide bonds per protein equivalent, was used as 8.6 mg equivalent/g protein, and α and β were 1.00 and 0.40.

#### 3.4.3. Molecular Weight Distribution

Size-exclusion high-performance liquid chromatography (SEC-HPLC) was performed to assess the peptide molecular weight distribution. Samples were analyzed using an AdvanceBio SEC LC column (130 Å, 7.8 × 150 mm, 2.7 µm) connected to a diode array detector (DAD) (Agilent Technologies, Santa Clara, CA, USA). A total of 5 μL of sample was injected onto the column kept at room temperature. The mobile phase consisted of pH 7 sodium phosphate buffer (150 mM). Samples were eluted at a flow rate of 0.5 mL/min and UV signal was measured at 220 nm. Each sample was filtered through a 0.45 μm PVDF filter. The AdvanceBio SEC 130 Å Protein Standard (Agilent Technologies, Santa Clara, CA, USA), composed of Ovoalbumin (45,000 Da), Myoglobin (17,000 Da), Aprotinin (6700 Da), Neurotensin (1700 Da) and Angiotensin II (1000 Da), was used to determine the elution time depending on the weight of the molecules. Every sample was diluted to a protein concentration of 3 g/L.

#### 3.4.4. Antioxidant Capacity Test

The ABTS (2,2-azinobis-(3-ethylbenzthiazoline-6-sulfonic acid)) assay adjusted to microplate volume was used to determine the antioxidant capacity [48]. The colorimetric results were measured in a Varioskan™ LUX multimode microplate reader (Thermo Fisher Scientific, Waltham, MA, USA). In brief, 7 mM ABTS solution and 2.45 mM potassium persulfate were diluted in PBS (phosphate-buffered saline) for an absorbance of approximately 0.7 at 734 nm. The determination consisted of the decrease in absorbance at 734 nm of the reagent solution, 6 min after the sample was added in the micro-well in a ratio of 1:100 (*v*/*v*) (sample: ABTS solution), as the result of the reduction of the radical colored ABTS. Trolox (Thermo Fisher Scientific, Waltham, MA, USA) was used as the standard, and the antioxidant capacity (AOC) was calculated as Trolox equivalent antioxidant capacity (TEAC, mg/g protein).

The antioxidant capacity yield (AOCy) represented the total release of TEAC (g) in each run of the experiment and was calculated by multiplying the AOC by the PEY (%), with the resulting value being expressed in TEAC g per g of protein in the initial sample.

AOCy = AOC × PEY(3)

#### 3.4.5. Antihypertensive Activity Assay

To evaluate the antihypertensive activity, the angiotensin-converting enzyme inhibition (ACEi) was measured by the fluorescence method developed by Sentandreu et al. [49] with some modifications [50].

In each well of a 96-well microplate reader (Thermo Fisher Scientific, Waltham, MA, USA), 40 µL of ultrapure water or ACE working solution was added and then made up to 80 µL by adding ultrapure water to blank, control or sample wells. ACE working solution was prepared by diluting ACE enzyme (angiotensin-converting enzyme, A6778–1 UN, Sigma-Aldrich) in a glycerol: water solution (50–50) to obtain a final ACE concentration of 1 U/mL. The enzyme reaction was initiated by adding 160 µL of substrate solution (3.6 mg of substrate (o-Abz-Gly-p-Phe(NO2)-Pro-OH) in 16 mL of Tris buffer 0.15 M pH 8.3) and incubating the mixture at 37 °C. After 30 min, fluorescence generated was measured using a Varioskan™ LUX multimode microplate reader (Thermo Fisher Scientific, Waltham, MA, USA) with excitation and emission wavelengths of 350 and 420 nm, respectively.

ACEi was expressed as the resulting inhibition for a dilution of 1 mg protein per milliliter. ACEy was the antihypertensive capacity yield obtained in the process and it was calculated by multiplying the ACEi by the PEY.

ACEy = ACEi × PEY (4)

### 3.5. Statistical Analysis

Experimental factors were analyzed via ANOVA (analysis of variance) and were considered significant when their probability (*p* value) was less than 0.05. Normal data distribution was verified after using the Shapiro–Wilk test and Levene’s test to assess the equality of variances. When equal variances were not assumed or data were not adjusted to a normal distribution, the Kruskal–Wallis statistic was used to compare the samples. Multiple comparisons were carried out using Tukey’s HSD test.

The statistical analysis of the model was performed using ANOVA. The adequacy of the model was determined by the coefficients of determination (R^2^) and adjusted R^2^ test. In the optimization trials, a univariant analysis between the bioactivities and the molecular weight (MW) ranges of the hydrolysates was performed using Spearmen correlation analysis. These correlation coefficients range from −1 to +1 and measure the strength of the linear relationship between the variables.

All statistical analyses were performed using the Statgraphics software (Statgraphics Centurion XVI software package, 16.2.04 version; Statgraphics Technologies, Inc., The Plains, VA, USA). This software was also used to perform the response surfaces.

## 4. Conclusions

A large number of hydrolysates using six different kinds of enzymes, with different specific activities, and different enzyme combinations and conditions, were produced and several positive results were obtained for the antihypertensive and antioxidant bioactivities.

Taking into account that the bioactivity tests were performed with raw hydrolysates, the obtained results are of great interest and were investigated within the WaSeaBi project in two steps, first an optimization of the process and second by scaling up the processes to better analyze yields and product characteristics.

The highest bioactivity values were obtained with the endoprotease of serine type at 70 and 50 °C, and with a broad-spectrum endoprotease, alone or with the combination of glutamic acid-specific protease and a blend of exopeptidases. These conditions led also to a higher degree of hydrolysis, which was subsequently corroborated by a positive correlation between the small molecular weight peptides (0.3–1 kDa) and the bioactivity yields.

Considering that the hydrolysis with the endoprotease of the serine type at 70 °C led to the best results in terms of protein extraction and bioactivities, it was selected for further process optimization.

The design of the experiment allowed the selection of the best operation conditions to maximize bioactivities and peptide yields. However, the resulting conditions differed and the definition of total antioxidant and antihypertensive yields allowed the maximizing of the overall process. A pilot trial in the optimized conditions led to a result in accordance with the developed model, which confirmed the technical feasibility of the process at industrial scale. As a conclusion, the production of bioactive peptides stands out as a promising solution for these fish by-catches.

## Figures and Tables

**Table 1:** Hydrolysis conditions in the initial screening.

Screening Run	Enzyme	Hydrolysis Conditions
S1	A: endoprotease of the serine type	1% 70 °C pH 9
S2	A: endoprotease of the serine type	1% 50 °C pH 6
S3	P: broad-spectrum endoprotease	1% 50 °C pH 6
S4	T: trypsin-specific protease	1% 45 °C pH 6
S5	C: chymotrypsin-like protease	1% 70 °C pH 6
S6	G: glutamic acid-specific protease	1% 50 °C pH 6
S7	A + F: endoprotease of the serine type + blend of endo- and exopeptidases	1% of each enzyme 50 °C pH 6
S8	P + F: broad-spectrum endoprotease + blend of endo- and exopeptidases	1% of each enzyme 50 °C pH 6
S9	P + G: broad-spectrum endoprotease + glutamic acid-specific protease	1% of each enzyme 50 °C pH 6
S10	CTR: without enzyme	50 °C pH 6

The enzyme concentration is relative to the protein content in the hydrolysis process. All the experiments were performed for 3 h.

**Table 2:** Box–Behnken experimental design, experimental runs and obtained values for the response variables.

BBD Run	Enzyme–Substrate Ratio (%)	Time (h)	Solid (%)	PEY (%)	DH (%)	AOC (mg TEAC/g)	AOCy (mg TEAC/g)	ACEi (% at 1 mg/mL)	ACEy (% at 1 mg/mL)
R1	2.00	4	50.0	61.64	7.94	160.79	99.11	21.50	13.25
R2	1.25	6	65.0	43.80	11.33	169.69	74.32	7.44	3.54
R3	0.50	4	50.0	43.30	8.11	174.96	75.75	6.74	2.92
R4	2.00	2	57.5	53.56	5.22	255.00	136.59	34.73	18.6
R5	0.50	4	65.0	35.89	9.90	125.02	44.87	24.50	8.79
R6	1.25	4	57.5	50.72	9.56	130.09	65.97	32.73	16.6
R7	1.25	2	65.0	44.97	4.32	125.97	56.66	23.62	10.62
R8	0.50	6	57.5	29.36	11.31	150.39	44.16	11.32	3.32
R9	1.25	6	50.0	49.09	12.64	150.15	73.71	16.53	8.12
R10	2.00	6	57.5	46.36	8.84	145.65	67.52	17.98	8.34
R11	1.25	2	50.0	54.71	4.57	150.88	82.54	33.59	18.38
R12	1.25	4	57.5	51.50	9.00	158.41	81.58	10.89	5.61
R13	1.25	4	57.5	42.16	6.74	142.71	60.16	19.01	7.59
R14	0.50	2	57.5	35.78	4.86	126.21	45.16	17.02	6.09
R15	2.00	4	65.0	54.27	11.50	191.14	103.72	17.76	9.64

**Table 3:** Reduced regression model coefficients and ANOVA significance levels of each term of the equation.

	Coefficients						*p*-Value					
	PEY	DH	AOC	ACEi	AOCy	ACEy	PEY	DH	AOC	ACEi	AOCy	ACEy
Intercept	281.496	40.849	535.329	−121.479	555.637	−6.323						
A: % Solids	−8.487	−1.586	−7.840	4.0604	−14.896	0.147	**0.0088**	0.4759	0.7126	0.8408	0.2842	0.3840
B: Time	1.954	5.201	−29.255	−3.568	−10.098	−3.385	**0.0434**	**0.0037**	0.5381	0.0596	0.2138	**0.0266**
C: % Enzyme	25.759	−3.354	−174.499	74.267	−41.763	39.325	**0.0001**	0.8955	**0.0345**	0.2263	**0.0059**	**0.0332**
AA	0.067	0.014		−0.026	0.090		**0.0431**	0.4210		0.7552	0.5522	
AB	0.074	−0.018	0.741		0.442	0.053	0.4727	0.7729	0.3682		0.4228	0.6911
AC		0.079	3.568	−0.956	1.577	−0.421		0.6326	0.1294	0.2527	0.2964	0.2567
BB	−0.937	−0.253	1.480		−0.617		0.0437	0.3146	0.6353	0.7961	0.7683	
BC		−0.472	−22.255	−1.842	−11.345	−1.248		0.4530	**0.0265**	0.5396	0.0753	0.3617
CC	−5.537	0.241	35.067	−2.623	11.712	−2.128	0.0806	0.8866	0.1472	0.7500	0.4432	0.5658
R^2^	94.10	85.70	79.65	61.44	86.76	72.76						
Adj R^2^	88.16	59.96	52.52	10.02	62.93	45.51						

PEY: protein extraction yield; AOC: antioxidant capacity; AOCy; antioxidant capacity yield; ACEi: antihypertensive capacity; ACEy: antihypertensive capacity yield. Data in bold are statistically significant factors (*p*-value < 0.05).

**Table 4:** Optimized conditions for each factor and the combination of optimized factors.

Dependent Variables	Solids (%)	Time (h)	Enzyme–Substrate Ratio (%)
PEY	50	3.07	2.0
DH	65.0	6.0	0.5
AOC	65.0	2.0	2.0
ACEi	50.0	2.0	2.0
AOCy	50.0	2.0	2.0
ACEy	50.0	2.0	2.0
AOCy + ACEy	50.0	2.0	2.0

PEY: protein extraction yield; DH: degree of hydrolysis; AOC: antioxidant capacity; AOCy; antioxidant capacity yield; ACEi: antihypertensive capacity; ACEy: antihypertensive capacity yield.

## Data Availability

The data are contained within the article.
